# Outflow Facility in Tube Shunt Fenestration

**DOI:** 10.5005/jp-journals-10028-1255

**Published:** 2018

**Authors:** Jessica Olayanju, Teresa Borras, Bahjat Qaqish, David Fleischman

**Affiliations:** 1 Department of Ophthalmology, University of North Carolina, Chapel Hill, North Carolina, USA; 2 North Carolina Translational and Clinical Sciences Institute, University of North Carolina, Chapel Hill, North Carolina, USA

**Keywords:** Aqueous shunt, Baerveldt, Fenestration, Venting slit

## Abstract

**Aim:**

Determination of the effect of varying fenestration technique, and simulated patch graft on outflow facility for Baerveldt tube.

**Materials and methods:**

Silicone tubing similar to Baerveldt implant (AMO, Santa Ana, CA) with different fenestrations techniques was connected to a digital manometer in a closed system with a fluid-filled syringe on a stand to adjust pressure. The venting slits included: (A) 4 piercings with 7–0 TG140-8 needle; (B) a 2-mm slit with a 15° blade; (C) 4 piercings with a 15° blade; (D) 9–0 Nylon on CS140-6 needle with suture stenting the fenestration.

**Results:**

For pressures of 10, 20, 30, 40 mm Hg in groups A to D, the average outflow facility (mL/min/mm Hg) were group A: 0.11, 0.20, 0.28, 0.40; group B: 0.30, 0.69, 0.98, 0.93; group C: 0.73, 0.80, 0.81, 0.88; group D: 0.58, 0.65, 0.80, 0.87. For external compression with 10 gram weights at pressures of 10, 20, 30, 40 mm Hg, outflow were group A: 0.0, 0.18, 0.20, 0.53; group B: 0.75, 0.70, 0.97, 1.21. Group C: 0.18, 0.03, 0.57, 0.04. Group D: 0.73, 0.90, 1.13, 0.91.

**Conclusion:**

Effectivity of venting slits in maintaining adequate IOP in the early postoperative period for non-valved glaucoma implant is variable, multifactorial and largely intraocular pressure (IOP) dependent.

**Clinical significance:**

This study explores methods of producing fenestration and the effects on outflow at different pressures in an attempt to determine which fenestration technique has more reproducible results that can be made applicable in clinical practice. This is also the first study to evaluate the effect of external pressures similar to scleral patch graft on the tube fenestrations.

**How to cite this article:**

Olayanju J, Borras T, Qaqish B, Fleischman D. Outflow Facility in Tube Shunt Fenestration. J Curr Glaucoma Pract 2018;12(3):113-118.

## INTRODUCTION

A recent paradigm shift in glaucoma management has placed aqueous humor drainage devices as an equivalent initial surgical option to trabeculectomy in uncontrolled glaucoma.^1–4^ Most frequently, these devices are implanted initially in the superotemporal quadrant. Almost all tube surgeries require a patch graft placed over the tube to prevent erosion of the overlying conjunctiva. Examples of glaucoma drainage devices include the Molteno^®^ 3 (Katena, New Jersey), Ahmed^®^ glaucoma valve (New World Medical Inc, Rancho Cucamonga, CA), and the Baerveldt^®^ glaucoma drainage device(Abbott Medical Optics Inc, Santa Ana, CA).

The Ahmed^®^ and Molteno^®^ have a built-in valve or flow-restrictor systems to immediately lower intraocular pressure. However, the surface area of the Ahmed and a lone Molteno 3 device are presumed to be a reason why they may have minor pressure reduction compared to the Baerveldt glaucoma drainage device.^5–8^ The Baerveldt implant is a non-valved glaucoma device that requires temporary ligation during the initial postoperative period to allow encapsulation of the plate which may take three to six weeks to occur. Inadequate encapsulation leads to hypotony. As opposed to valved implants, the Baerveldt implant does not have an inherent system to control intraocular pressure which may be problematic in patients who require immediate pressure reduction. Venting slits or suture stents can be placed in the tubing anterior to the ligature to help lower IOP, but their effect can be highly variable.^9–16^ It is not uncommon that fenestrations have anywhere from the negligible effect on IOP to resulting in frank hypotony from over-filtration.

We hypothesize that one of the reasons for the unpredictable nature of fenestrations is due in part to the patch graft placed on top of the tube, which may compress the slits on the lateral aspect of the tubing. This may be dependent on the amount of pressure applied to the tube from the overlying patch graft. The purpose of this study is to determine the effect of venting slit techniques on fluid egress at various IOP and different external pressures to simulate the scleral patch graft.

## MATERIALS AND METHODS

**Figs 1A and B:**
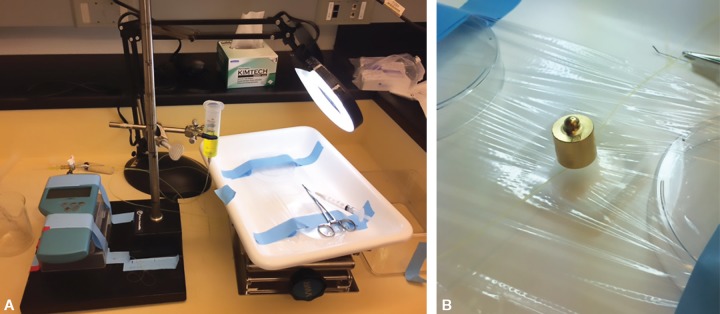
(A) 3-way stop cock connected to silicone tubing, a digital manometer, and an adjustable open reservoir bottle; (B) 10 g brass weight overlying fenestrated tubing on a plastic wrap

Soft, non-sterile silicone tubing (Instech Labs, silicone infusion tubing Model BTSIL-025) with external diameter of 0.635 mm and internal diameter of 0.305 mm closely meeting the specifications of silicone tubing incorporated into the Baerveldt implant (0.63 mm × 0.30 mm) was connected at one end to a cannula via a 3-way stopcock to both a digital manometer (Omega DPI 705) and an adjustable open reservoir bottle. The hydrostatic pressure in the system was then slowly elevated until fluorescein-stained water flowed from the valve and adjusted to various heights for a controlled IOP measured with the digital manometer. The tubing was then clamped off on the other end using a hemostat to create a closed system (Fig. 1A). The tubing was taped in a manner to not stretch and distort the tubing, leaving it just taut enough to simulate the tubing *in vivo*.

The IOP was held constant during each experimental measurement group. The manometer-confirmed pressures were increased in 10 mm Hg increments from 10 to 40 mm Hg. At each increment, 3 measurements were taken using different tubing with the same technique. A plastic weighing dish was placed below the tubing with the venting slits to collect any egressed fluid at a set time of 2 minutes. The various venting slits included: (A) 4 piercings with 7-0TG140-8 needle; (B) a single 2 mm longitudinal slit (measured with calipers) along the lateral walls of the tubing performed with a 15° blade penetrating both walls of the tubing; (C) 4 piercings with the 15° blade (full thickness through both walls of the tubing); (D) 9-0 nylon on CS140-6 needle with suture stenting the slit, in a manner described by Dr. James Brandt, but with nylon instead of vicryl. The fenestrations were performed by two of the authors (DF, JO). DF trained JO on each technique of fenestration before performing. Each fenestration was performed or observed by DF.

A digital timer was set for 2 minutes and started after flow was confirmed through the fenestration. All fluid was cleaned and wiped away prior to starting the timer. The fluid was collected in the plastic tray, and at the conclusion of two minutes, any remaining fluid remaining on the outside of the tubing due to surface tension was gently teased into the plate. The exact volume of fluid was measured using Gilson micropipette systems to the nearest microliter.

To measure the effect of external pressure on each of these venting slit techniques, the tubing was placed on plastic wrap and a 10 gram (g) brass weight was placed on the tubing at the area of the venting slits to simulate a patch graft, with an effort to allow the weight to distribute evenly as possible on the tubing (Fig. 1B). Egressed fluid was collected after 2 minutes at induced pressure increments of 10, 20, 30, and 40 mm Hg. The procedure was performed with a 20 g brass weight. The exact volume of the fluid was measured using the Gilson micropipette system.

The data were analyzed with a square root transformation of flow per minute to allow for the variance to be approximately constant over different pressures.

## RESULTS

In group A (4 piercings with 7-0 TG140-8 needle), the mean outflow facility (mL/min/mm Hg) was 0.11, 0.20, 0.28, and 0.40 for pressure of 10, 20, 30 and 40 mm Hg respectively. In group B the mean outflow facility was 0.30, 0.69, 0.98, and 0.93 for pressures of 10, 20, 30, and 40 mm Hg respectively. In group C, the mean outflow facility was 0.73, 0.80, 0.81, and 0.88 for pressure of 10, 20, 30, and 40 mmHg, respectively. For group D, the mean outflow facility was 0.58, 0.65, 0.80, and 0.87 for pressure of 10, 20, 30 and 40 mm Hg, respectively. There was an increase in the outflow facility with incremental increase in simulated intraocular pressure. This trend for flow to increase with pressure is depicted in Graph 2.

**Graphs 1A to D:**
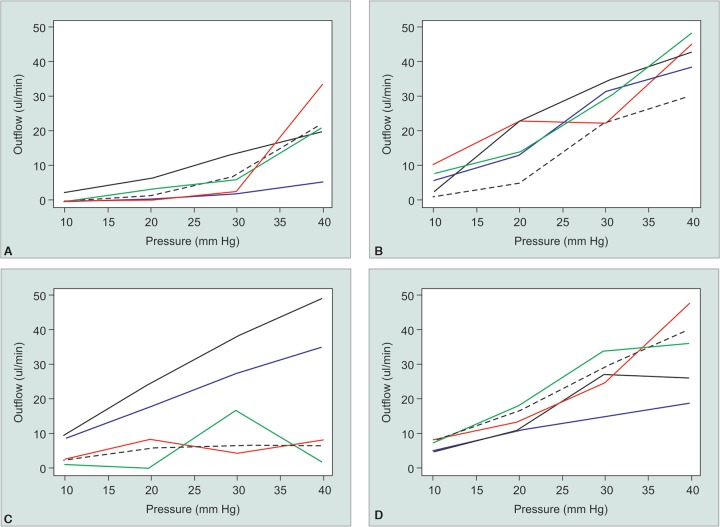
Outflow facility through the tube fenestrations with (green and red) and without (black solid) external pressures. (A) 4 piercings with 7-0 TG 140-8 needle, 10 g (green), 20 g (red); (B) 2 mm slit with 15° blade, 10 g (green), 20 g (red); (C) 4 piercings with 15° blade, 10 g (green), 20 g (red); (D) 9-0 Nylon on CS 140-6 needle with suture stenting, 10 g (green), 20 g (red)

The outcomes for the simulated patch graft with 10 and 20 g weights were highly variable and do not seem to make a difference, except perhaps in group B (Graph 1). Upon compression of Fenestrated A tube with 10 g weight, the outflow (mL/min/mm Hg)changed from 0.00, 0.08, 0.25, and 0.56 without compression to 0.00, 0.18, 0.20, 0.53 for pressures of 10, 20, 30, and 40 mm Hg respectively, and then to 0.00, 0.00, 0.09, and 0.84 with 20 g weight at respective pressures (*p* = 0.45).

In Fenestration B, the outflow facility increased with the 10g and 20 g weight at low pressures of 10 and 20 mm Hg but did not have a large effect at higher pressures above 30 mm Hg. For instance, the initial outflow without compression were 0.25, 1.15, 1.14, 1.07 at pressures of 10, 20, 30, and 40 mm Hg, respectively, then changed to 0.75, 0.70, 0.97, 1.21 with 10 g weight (*p* = 0.49) then 1.05, 1.15, 0.74, and 1.13 with 20 g weight (*p* = 0.34).

For fenestration C, the weight compression decreased the outflow facility at all the pressures measured. Initial outflow without compression were 1.00, 1.23, 1.27, and 1.23 at pressures of 10, 20, 30, 40 mm Hg, respectively, then decreased to 0.18, 0.03, 0.57, and 0.04 with 10 g weight (*p* = 0.37), then 0.28, 0.41, 0.16, and 0.21 with 20 g weight (*p* = 0.38).

With the fenestration D group, the weight compression increased the outflow facility with 20 g but remained the same with 10 g. Initial outflow without compression were 0.78, 0.83, 0.98, and 1.01 at pressures of 10, 20, 30, 40 mm Hg. The outflow with 10 g were 0.73, 0.90, 1.13, and 0.91 (*p* = 0.37), while the results with 20 g were 1.55, 1.58, 1.96, and 2.11 at set pressures thus allowing more fluid egression through the fenestrated tubes (*p* =0.00087).

## DISCUSSION

In a laboratory setting, the amount of fluid egress from any fenestration technique is proportional to the simulated intraocular pressure. While no technique was found to be considerably superior amongst the group, the suture-stenting technique (fenestration type D) did produce a more consistent fluid egress than all other techniques. External force over the fenestrations did not produce a consistent reduction in fluid egress from the fenestrations, and in fact sometimes increased outflow, disproving our hypothesis that compression of a horizontal slit would result in the decreased outflow. We offer a theory below as to why this was the case. However, only the suture-stenting technique did produce a statistically significant increase in outflow with the 20 g simulated patch.

In order to immediately control intraocular pressures following placement of a ligated, non-valved glaucoma drainage device, a variety of techniques to fenestrate a tube anterior to the ligature have been developed. This method has been found, in some studies, to adequately control IOP in the immediate postoperative period.^12–14^ Other studies^10,11^ indicate significant variability, however. Further, complications such as hypotony may result from the fenestrations.

We found variable results with the four different techniques and even variability within the same technique during 3 measurements with different tubing. In general, the higher the simulated intraocular pressure, the higher the outflow facility. The 10 g and 20 g weights did not influence outflow much, except by magnitude in group B (2 mm 15° blade) and by statistical significance in group D (suture stent). The flow of fluid through the fenestrations overall depended on the hydrostatic pressure as initially depicted in a similar laboratory investigation by Brooks et al.,^17^ which concluded that a longitudinally fashioned 2.0 mm fenestration proximal to an external occluding ligature produced a reliable opening pressure in the range of 4.6 to 17.7 mm Hg. This study also noted the lack of reproducibility especially with smaller fenestrations as well as larger fenestrations and postulated that factors other than hydrostatic pressure are involved in determining the outflow facility and opening pressure of the slit, which they likened to a valve.

Trible and Brown^13^ retrospectively evaluated the placement of an occlusive 7–0 polyglactin suture just anterior to the plate followed by a through-and-through penetration of the tube anterior to the occlusive ligature with a standardized 15° blade in 51 eyes. They concluded that this modification provides adequate IOP control in the early postoperative period as did Arismendi^12^ who titrated the absorbable ligature to allow slow flow from postoperative day 1 while avoiding hypotony. Emerik^14^ retrospectively reviewed patients who underwent Baerveldt implantation with and without fenestration at 1 year and reported a statistically significant decrease in IOP within the first month of surgery in 69 eyes that underwent 2 or 3 through-and-through fenestrations of the tube with suture TG-140 needle when compared to 42 patients without fenestrations. In the prospective TVT study^,16^ 82 of the 107 Baerveldt tubes underwent fenestrations, in which 56 patients had 1–4 suture needle fenestrations and 26 patients with 1–5 surgical blade fenestrations. They reported similar IOP reduction and rates of early hypotony with fenestrations performed using a needle and blade; thus no difference was noted in outcomes with either technique. A recent retrospective study by Yadgarov et al.^18^ reviewed 119 eyes with a single tube fenestration stented with 10–0 polyglactin suture anterior to the occlusive ligature after Baerveldt surgery. They report a significant reduction in IOP and glaucoma medications 3 weeks postoperatively during the period tube occlusion and low incidence of hypotony-related complications. The pressure range in all these studies was 20 to 40 mm Hg especially in the fenestrated groups, and although the results largely show a reduction in IOP, there were reports of hypotony as well, displaying variable IOP outcomes regardless of fenestration techniques.

**Figs 2A to F:**
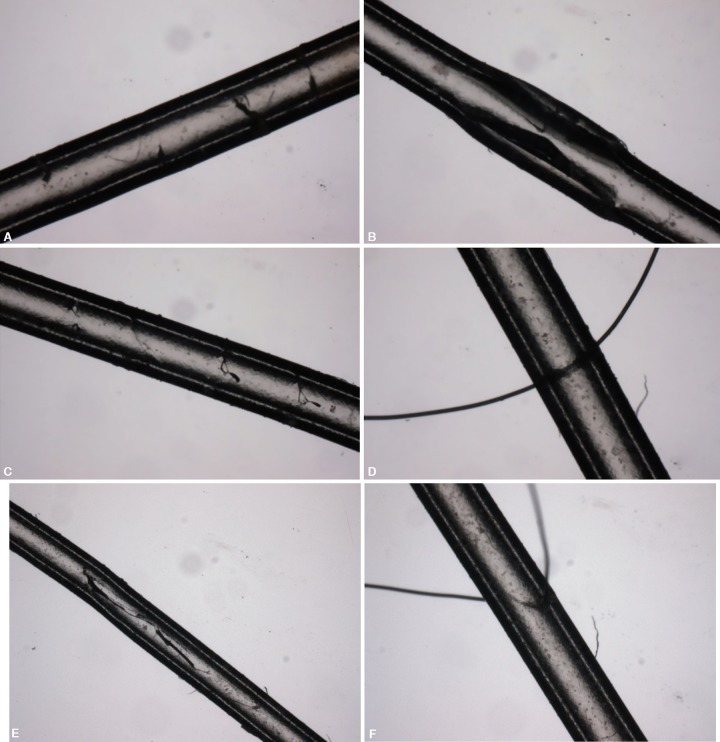
Microscopic image of fenestration types: (A) 4 piercings with 7-0 TG 140-8 needle; (B) Single 2 mm full thickness longitudinal slit with a 15° blade; (C) Full-thickness piercings with the 15° blade; (D) 9-0 Nylon on CS 140-6 needle with suture stenting the slit; (E) side view of “B”; (F) side view of “D”

In our study, external compression has a compressive effect in all four fenestration techniques at an IOP of 10 mm Hg. Thereafter, the effect of the 10 g and 20 g weight varies considerably. We suspect that this depends on the actual microarchitecture of the fenestration. Since these fenestrations are created manually, in a technique similar to what is performed intraoperatively under a microscope, the slits may be imperfect and likely not truly horizontal. This is consistent with the microscopic appearance of the different techniques of fenestration (Fig. 2). While the approach was intended to be purely horizontal, the microstructure reveals otherwise. Perhaps in certain imperfect fenestrations, weights may, in fact, create a gape in the fenestration, while in other cases the external weight reinforces the slits and closes them, necessitating a higher intraluminal hydrostatic pressure in order to break through the valve-like mechanism.

The results of this study could be extrapolated as follows. With higher intraocular pressures, there is likely to flow through the fenestrations in most circumstances until the intraocular pressure declines to a value where the pressure differential within the tubing is either equilibrated with the tissue pressure outside of the tube, or insufficient to penetrate the slit-like valve of the fenestration depending on its construction and the pressure induced by the patch graft. When intraocular pressure increases reopening the valve, the flow rate is likely reduced in a manner consistent with the results of our investigation. The reduced flow rates would permit for scarring and encapsulation around the tubing and fenestrations, eventually sealing the fenestration and resulting in elevated intraocular pressures prior to the release of ligation. This further complicates and underscores the dynamic processes that occur in the post-operative state. A stent across the fenestration, such as the 9–0 Vicryl or any other suture, by providing a wick, may be effective in a continuous flow and controlling intraocular pressure, although this technique is still subject to fluid control variability. It is important to note that the present study is testing the mechanical properties of the fenestrated tube at each pressure point, and not as an *in vitro* simulation of fenestrated tube function in vivo. This is because as fluid leaves the tube, IOP will incrementally decrease creating dynamic and different stress on the intraluminal and valvular component of the tube. By controlling and maintaining IOP, the study is minimizing perturbations on the system to allow elucidation of the outflow properties of the fenestrated tube at each pressure point.

Limitations of this study include the difference of surgeons performing the fenestrations, although each tube fenestration was performed or observed by the same ophthalmologist (DF). Techniques were not different between the two surgeons. The devices used were similar in consistency and dimensions to the Baerveldt tubes, but they were not the same, and therefore any important differences in mechanical structure could affect measurements. We are unaware of any measurement of the amount of force applied by suturing a patch graft and closing the conjunctiva over the graft and fenestrated tubing; therefore a 10 g and 20 g weight may be inadequate representations of the amount of force applied in *in vivo* conditions.

## CONCLUSION

The venting slits for a ligated tube similar in specifications to a Baerveldt implant provided highly variable results within each technique at different IOP measurements as well as with external pressure, simulating the compression induced by a patch graft. Effectivity of venting slits in maintaining adequate IOP in the early postoperative period for non-valved glaucoma implant is multifactorial and largely dependent on preoperative IOP.

## CLINICAL SIGNIFICANCE

The use of fenestrations in a ligated tube shunt is a useful adjunct to Baerveldt surgery allowing for immediate reduction of IOP, however the IOP outcomes are often variable. This study explores methods of producing fenestration and the effects on outflow at different pressures in an attempt to determine which fenestration technique has more reproducible results that can be made applicable in clinical practice. This is also the first study to evaluate the effect of external pressures similar to scleral patch graft on the tube fenestrations.
